# Severe COVID-19 in Uganda across Two Epidemic Phases: A Prospective Cohort Study

**DOI:** 10.4269/ajtmh.21-0551

**Published:** 2021-08-09

**Authors:** Barnabas Bakamutumaho, Matthew J. Cummings, Nicholas Owor, John Kayiwa, Joyce Namulondo, Timothy Byaruhanga, Moses Muwanga, Christopher Nsereko, Emmanuel Rwamutwe, Roselyn Mutonyi, Josephine Achan, Lucy Wanyenze, Alice Ndazarwe, Ruth Nakanjako, Richard Natuhwera, Annet Nsangi, Henry Kyobe Bosa, Felix Ocom, Max R. O’Donnell, Bernard Kikaire, Julius J. Lutwama, Collaboration for Clinical and Laboratory Characterization of COVID-19 in Uganda

**Affiliations:** 1 Uganda Virus Research Institute, Entebbe, Uganda;; 2 Vagelos College of Physicians and Surgeons, Columbia University, New York, New York;; 3 Mailman School of Public Health, Columbia University, New York, New York;; 4 Entebbe Regional Referral Hospital, Entebbe, Uganda;; 5 Uganda Peoples’ Defence Force, Kampala, Uganda;; 6 Ministry of Health, Kampala, Uganda;; 7 Makerere University College of Health Sciences, Kampala, Uganda

## Abstract

Among a prospective cohort of children and adults admitted to a national COVID-19 treatment unit in Uganda from March to December 2020, we characterized the epidemiology of and risk factors for severe illness. Across two epidemic phases differentiated by varying levels of community transmission, the proportion of patients admitted with WHO-defined severe COVID-19 ranged from 5% (7/146; 95% CI: 2–10) to 33% (41/124; 95% CI: 25–42); 21% (26/124; 95% CI: 14–29%) of patients admitted during the peak phase received oxygen therapy. Severe COVID-19 was associated with older age, male sex, and longer duration of illness before admission. Coinfection with HIV was not associated with illness severity; malaria or tuberculosis coinfection was rare. No patients died during admission. Despite low mortality, hospital incidence of severe COVID-19 during the first epidemic peak in Uganda was substantial. Improvements in vaccine deployment and acute care capacity, including oxygen delivery, are urgently needed to prevent and manage severe COVID-19 in sub-Saharan Africa.

## INTRODUCTION

Little is known about the clinical epidemiology of severe COVID-19 in sub-Saharan Africa (SSA). In Uganda, where SARS-CoV-2 was imported via travel in March 2020, over 80,000 cases have been reported.^1^^–^^3^ Similar to other settings, distinct epidemic phases of COVID-19 have been observed in Uganda, differentiated by scenarios of sporadic cases followed by community transmission and hospital surges.^4^ Here, we characterize the epidemiology of and risk factors for severe COVID-19 among a prospective cohort of children and adults admitted to a national COVID-19 treatment unit in Uganda during the first two phases of the epidemic.

## METHODS

This prospective observational study was conducted at Entebbe Regional Referral Hospital (ERRH), a 200-bed public hospital in central Uganda, from March 22 to December 31, 2020. During this period, ERRH was designated as a national isolation and treatment unit; only patients with COVID-19 were admitted to the unit. There is no intensive care unit or piped oxygen available at ERRH. Oxygen concentrators with capacity to provide 4–6 L/minute of oxygen, typically via nasal cannula, were provided as part of the study program.

Patients were included in this study if they fulfilled the following criteria: 1) were admitted to the ERRH COVID-19 treatment unit during the study period, 2) ≥ 5 years of age, 3) had laboratory-confirmed SARS-CoV-2 infection, and 4) were able to provide informed consent or had a surrogate, parent, or guardian available to do so. Pregnant women were excluded. Testing for SARS-CoV-2 was performed on naso- and/or oro-pharyngeal swab samples at Uganda Virus Research Institute or other Ministry of Health–certified testing centers using polymerase chain reaction (Charité, Berlin, Germany; DaAnGene Co. Ltd, Guangzhou, China).

At enrollment, clinical assessments were performed, and data were recorded using a modified form developed by the International Severe Acute Respiratory and Emerging Infection Consortium and WHO. For all patients, rapid testing was performed for malaria and HIV. For HIV-infected patients, testing for tuberculosis (TB) was performed if samples were obtainable. Details on pathogen diagnostics are provided in the supplemental materials.

We divided the study period into two phases because criteria for admission to the COVID-19 unit at ERRH, defined by Ministry of Health guidelines, were modified over time in response to evolving case surges.^5^ These guidelines set thresholds for admission based on bed surge capacity across isolation and treatment units nationwide.^5^ When ≥ 60% of bed capacity dedicated to COVID-19 management was available (approximately March–July 2020, a period with more sporadic cases and less community transmission; study phase 1), all cases of SARS-CoV-2 infection, regardless of severity, were admitted to isolation and treatment units. When this capacity was reached (approximately August—December 2020, a period with more community-transmission; study phase 2), admission was reserved for patients with or considered at risk for severe infection.^5^

Throughout admission, all management decisions were made by hospital clinicians independent of the study team and were informed by national COVID-19 treatment guidelines.^3^^,^^4^ The study team recorded management details until discharge, transfer to another health facility, or death.

The primary outcome of this study was a diagnosis of severe COVID-19. Based on criteria established by WHO, we considered patients to have severe COVID-19 if they fulfilled at least one of the following criteria during admission: 1) oxygen saturation < 90% on room air, 2) respiratory rate > 30 breaths/minute, 3) showed signs of respiratory distress (chest in-drawing, nasal flaring, or grunting respirations), or 4) received oxygen therapy.^6^ Secondary outcomes included a composite measure of in-hospital outcome (death in-hospital or transfer to Uganda’s national referral hospital due to progressive illness severity) and functional status at discharge, measured using Lansky or Karnofsky Performance Status.

Given differences in admission criteria across the two study phases, we performed our analyses in the general study population (primary analysis) and stratified by phase (secondary analysis). Continuous variables were expressed as medians (interquartile range), and categorical variables were summarized as frequencies and percentages with 95% CIs provided for outcome data. In the primary analysis, factors associated with severe (versus mild–moderate) COVID-19 were determined using Wilcoxon rank sum, Fisher’s exact, or χ^2^ tests, with two-sided *P* values ≤ 0.05 considered significant. Analyses were performed using Stata (v16, StataCorp, College Station, TX).

Each enrolled participant, their surrogate, or parent/guardian provided written informed consent. The study was approved by ethics committees at Columbia University, Uganda Virus Research Institute, and Uganda National Council for Science and Technology.

## RESULTS

A total of 270 patients were enrolled during the study period (Supplemental Table 1; Supplemental Figure 1). The median age was 35 years (interquartile range: 27–43); 83% were male. Cough, fever, and headache were the most common symptoms. Ten percent and 1% had HIV and malaria coinfection, respectively. None of the patients tested had TB coinfection.

**Figure 1.**
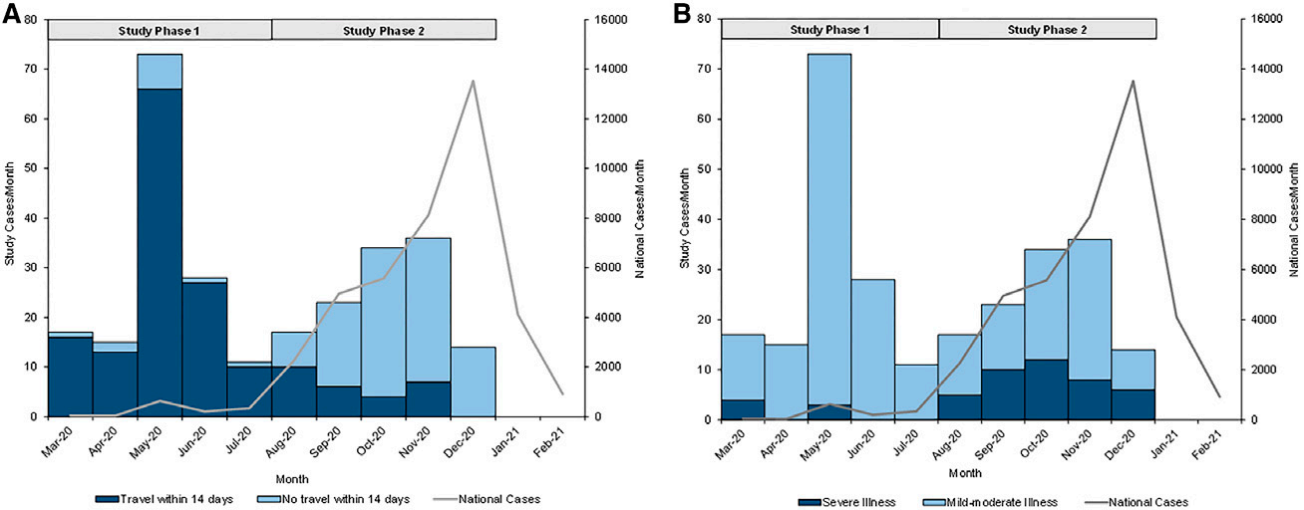
Epidemiologic curve of enrolled patients, stratified by (**A**) travel history and (**B**) illness severity and plotted with national COVID-19 case counts. National COVID-19 case counts were obtained from the WHO COVID-19 Uganda Dashboard.^7^ Study phase 1: March–July 2020; study phase 2: August–December 2020. This figure appears in color at www.ajtmh.org.

One hundred forty-six (54%) and 124 (46%) patients were admitted during study phases 1 and 2, respectively (Table 1). Patients admitted during phase 1 were more likely to be male and to have recently traveled (Figure 1A). Patients admitted during phase 2 were more likely to be healthcare workers and to have hypertension and presented later in their illness course.

**Table 1:** Characteristics of enrolled patients stratified by study phase

Patient characteristic	Phase 1 (*N* = 146)	Phase 2 (*N* = 124)	*P* value*
Male sex, *n* (%)	132/146 (90)	93/124 (75)	0.001
Age, years, median (IQR)	34 (27–42)	36 (28–46)	0.332
Healthcare or laboratory worker, *n* (%)	0/146 (0)	10/124 (8)	< 0.001
Travel within 14 days prior to admission, *n* (%)	134/146 (92)	27/124 (22)	< 0.001
Co-existing hypertension, *n* (%)	3/146 (2)	22/124 (18)	< 0.001
Symptoms reported, *n* (%)			
Cough	36/146 (25)	74/124 (60)	< 0.001
Fever	34/146 (23)	44/124 (35)	0.028
Headache	23/146 (16)	39/124 (31)	0.002
Rhinorrhea	28/146 (19)	32/124 (26)	0.190
Shortness of breath	2/146 (1)	34/124 (27)	<0.001
Sore throat	12/146 (8)	17/124 (14)	0.150
Diarrhea	4/146 (3)	7/124 (6)	0.355
Mildly symptomatic or asymptomatic,¶ *n* (%)	66/146 (45)	23/124 (19)	< 0.001
Duration of illness prior to hospitalization, days, median (IQR)†	4 (2–5)	6 (3–7)	0.026
Vital signs			
Temperature ≥ 38°C, *n* (%)	8/146 (5)	7/124 (6)	0.953
Heart rate, beats/min, median (IQR)	80 (73–90)	90 (81–102)	< 0.001
Respiratory rate, breaths/min, median (IQR)‡	18 (18–20)	18 (18–22)	0.448
Systolic blood pressure, mm of Hg, median (IQR)	120 (110–129)	124 (116–133)	0.001
Oxygen saturation, %, median (IQR)	97 (96–98)	97 (95–98)	0.284
Glasgow Coma Score, median (IQR)	15 (14–15)	15 (14–15)	0.025
Unable to ambulate without assistance, *n* (%)	0/146 (0)	11/124 (9)	< 0.001
Mid-upper arm circumference, mm, median (IQR)§	20 (18–23)	22 (20–24)	< 0.001
Coinfections, *n* (%)			
Malaria	3/146 (2)	1/124 (1)	0.697
HIV	18/146 (12)	8/124 (6)	0.103
Microbiological TB‖	0/16 (0)	0/5 (0)	–
Clinical management, *n* (%)			
Received oxygen therapy	0/146 (0)	26/124 (21)	< 0.001
Received antibacterial agent	75/146 (51)	96/124 (77)	< 0.001
Received HCQ or chloroquine	28/146 (19)	0/124 (0)	< 0.001
Received corticosteroids	0/146 (0)	38/124 (31)	< 0.001
Patient outcomes, *n* (%)			
Severe COVID-19	7/146 (5)	41/124 (33)	< 0.001
Died in-hospital	0/146 (0)	0/124 (0)	–
Transferred to national referral hospital	0/146 (0)	1/124 (0.8)	0.459
Karnofsky or Lansky score ≥ 80 at discharge	143/143 (100)	123/124 (99)	0.464

HCQ = hydroxychloroquine; IQR = interquartile range, TB = tuberculosis.

* Wilcoxon rank-sum, Fisher’s exact or χ^2^ test.

† Known for 135 patients.

‡ Known for 255 patients.

§ Known for 214 patients.

‖ Denominator of patients who underwent TB testing (urine TB-LAM or sputum Xpert MTB/RIF Ultra or smear). ¶Defined as absence of reported cough, fever, headache, rhinorrhea, shortness of breath, sore throat, diarrhea, or night sweats.

In the general study population, 18% (95% CI: 13–23) of patients met the primary outcome of WHO-defined severe COVID-19 (Supplemental Table 1). This included 5% (95% CI: 2–10) and 33% (95% CI: 25–42) of patients admitted during study phases 1 and 2, respectively (Table 1, Figure 1B). In the general population, severe COVID-19 was significantly associated with older age, male sex, and a longer duration of illness prior to admission (Table 2). These associations were driven by patients admitted during phase 2 (Supplemental Tables 2 and 3). Across and within each phase, we observed no significant associations between illness severity and HIV coinfection. One patient in phase 2 required transfer to the national referral hospital; no patients died in-hospital (Table 1).

**Table 2:** Characteristics of patients with and without severe COVID-19 across both study phases

Patient characteristic	Severe illness (*N* = 48)	Mild-moderate illness (*N* = 222)	*P* value*
Male sex, *n* (%)	45/48 (94)	180/222 (81)	0.033
Age, years, median (IQR)	41 (35–50)	33 (27–41)	0.0001
Co-existing hypertension, *n* (%)	5/48 (10)	20/222 (9)	0.784
Symptoms reported, *n* (%)			
Cough	33/48 (69)	77/222 (35)	< 0.0001
Fever	27/48 (56)	51/222 (23)	< 0.0001
Headache	9/48 (19)	53/222 (24)	0.444
Rhinorrhea	4/48 (8)	56/222 (25)	0.011
Shortness of breath	22/48 (46)	14/222 (6)	< 0.0001
Sore throat	6/48 (13)	23/222 (10)	0.664
Diarrhea	3/48 (6)	8/222 (4)	0.419
Duration of illness prior to hospitalization, days, median (IQR)†	6 (3–7)	4 (2–7)	0.009
Vital signs			
Temperature ≥ 38°C, *n* (%)	7/48 (15)	8/222 (4)	0.008
Heart rate, beats/min, median (IQR)	91 (79–104)	83 (76–93)	0.002
Respiratory rate, breaths/min, median (IQR)‡	22 (19–24)	18 (18–20)	–
Systolic blood pressure, mm Hg, median (IQR)	126 (112–132)	120 (114–130)	0.373
Oxygen saturation, %, median (IQR)	95 (92–98)	97 (96–98)	–
Glasgow Coma Score, median (IQR)	15 (14–15)	15 (14–15)	0.189
Unable to ambulate without assistance, *n* (%)	10/48 (21)	1/222 (0.5)	< 0.0001
Mid-upper arm circumference, mm, median (IQR)§	22 (20–24)	21 (19–23)	0.309
Coinfections, *n* (%)			
Malaria	0/48 (0)	4/222 (2)	–
HIV	5/48 (10)	21/222 (9)	0.791
Microbiological TB‖	0/2 (0)	0/19 (0)	–
Clinical management, *n* (%)			
Received oxygen therapy	26/48 (54)	0/222 (0)	–
Received antibacterial agent	40/48 (83)	131/222 (59)	0.002
Received HCQ or chloroquine	2/48 (4)	26/222 (12)	0.189
Received corticosteroids	26/48 (54)	12/222 (5)	< 0.0001

HCQ = hydroxychloroquine; IQR = interquartile range; TB = tuberculosis.

* Wilcoxon rank-sum, Fisher’s exact, or χ^2^ test.

† Known for 135 patients.

‡ Known for 255 patients.

§ Known for 214 patients.

‖ Denominator of patients who underwent TB testing (urine TB-LAM or sputum Xpert MTB/RIF Ultra or smear).

During admission, 10% of patients received oxygen therapy, delivered via concentrators and cylinders, including 21% of patients admitted during phase 2 (Table 1, Supplemental Table 1). Patients admitted during phase 1 were more likely to receive hydroxychloroquine or chloroquine, whereas those admitted during phase 2 were more likely to receive corticosteroids. Among patients with severe illness admitted during phase 2, those who received corticosteroids were older and had more deranged vital signs (Supplemental Table 4). Nearly all patients administered corticosteroids received oxygen therapy and antibacterial agents.

## DISCUSSION

Among a prospective cohort of patients admitted to a national COVID-19 treatment unit in Uganda during the first two phases of the epidemic, the incidence of WHO-defined severe COVID-19 ranged from 5% to 33%. Consistent with data from high-income countries, older age and male sex were associated with illness severity.^8^^,^^9^ Although HIV and TB coinfections have been associated with COVID-19–related mortality elsewhere in the region,^10^ these coinfections were infrequent in our cohort, as was malaria. Similar to a recent study from Malawi, we observed no significant association between HIV coinfection and illness severity.^11^ Given these likely context-specific findings, continued efforts are needed to elucidate the epidemiology of severe COVID-19 in SSA.

During the peak epidemic phase, over 20% of patients received oxygen therapy and 63% with severe illness received corticosteroids. Because administration of oxygen is an essential treatment of all severe respiratory infections, expanded access to oxygen therapy in SSA is a global imperative.^12^ In the interim, efforts are needed to optimize targeted delivery of oxygen therapy, corticosteroids, and other evidence-based treatments to severely ill patients in the region.

Despite substantial incidence of severe COVID-19 during the epidemic peak, no patients died in-hospital, and only one was transferred to the national referral hospital due to progressive illness severity. This finding is largely consistent with data reported across Uganda, where the national case fatality rate for SARS-CoV-2 infection during the study period was 0.7%.^1^ Although under-reporting is possible and reasons for low mortality remain uncertain, a relatively young population, lower prevalence of high-risk comorbidities, and meteorological variables have been proposed.^13^^,^^14^ Nonetheless, as COVID-19 incidence and mortality in SSA are rising substantially, improvements in vaccine deployment and acute care capacity remain urgently needed to avoid preventable mortality.^15^

This study has limitations. First, our findings were derived from a single-center study. Second, the frequency of HIV, TB, and malaria was low, under-powering comparisons between these coinfections and outcomes. Third, our protocol excluded pregnant women, who are known to have higher risk of severe COVID-19.^16^ Fourth, we only obtained in-hospital outcomes. Although nearly all patients were discharged alive with normal functional status, future studies are needed to characterize longer-term outcomes and functional sequelae of COVID-19 in SSA.

Despite low mortality, hospital incidence of severe COVID-19 during the first epidemic peak in Uganda was substantial. Improvements in vaccine deployment and acute care capacity are urgently needed to prevent and manage severe COVID-19 in SSA.

## Supplemental materials


Supplemental materials
