# Supplementary material for: Severe COVID-19 in Uganda across Two Epidemic Phases: A Prospective Cohort Study
Source: Am J Trop Med Hyg. 2021 Aug 9;105(3):740–4. doi: 10.4269/ajtmh.21-0551 (PMC8592357; doi:10.4269/ajtmh.21-0551)

**Supplementary Material**

Pathogen Diagnostics

For all enrolled patients, rapid testing was performed for malaria and HIV; for HIV-infected patients testing for tuberculosis (TB) was also performed if samples could be obtained. Results were communicated to treating clinicians as soon as possible. For malaria, rapid testing was performed using qualitative detection of histidine‐rich protein II and lactate dehydrogenase of *P. falciparum* in whole-blood using the SD Bioline Malaria AG P.f. platform (Alere/Abbott, Abbott Park, IL, USA). For all patients not known to be HIV infected, HIV testing was performed using serial diagnostic platforms (Determine HIV-1/2 Ag/Ab, Alere/Abbott; Chembio HIV 1/2 Stat-Pak, Chembio Diagnostic Systems, Medford, NY, USA; Uni-gold Recombigen HIV-1/2, Trinity Biotech, Ireland). For enrolled HIV-infected patients (known or newly diagnosed), a single urine sample obtained via spontaneous void and a single spontaneously expectorated sputum sample, if obtainable, were tested for evidence of *Mycobacterium tuberculosis* (MTB) infection by the study laboratory technician. For urine samples, 60μL of unconcentrated urine was tested using the Determine™ TB-LAM Ag assay (Alere/Abbott) as per the manufacturer’s recommended operating procedure. The intensity of any visible band on the test strip was graded by comparing it with band intensities on the manufacturer’s post-2014 reference card scale; results were considered positive using the grade 1 cutoff. For sputum samples, testing was performed using the Xpert MTB/RIF Ultra platform (Cepheid, Sunnyvale, CA, USA). Sputum smear microscopy was performed at the discretion of treating clinicians as was Xpert MTB/RIF Ultra testing of sputum for non-HIV-infected patients.

**Supplemental Tables**

**Table S1: Characteristics of patients in the general study population**

| **Patient characteristic** | **Study Population**  **N=270**  **N (%)** |
| --- | --- |
| Male sex, n (%) | 225/270 (83) |
| Age group, years, n (%)  5-11  12-17  18-24  25-34  35-44  45-54  55-64  ≥65  Age, years, median (IQR) | 9/270 (3)  2/270 (1)  25/270 (9)  96/270 (36)  78/270 (29)  40/270 (15)  18/270 (7)  2/270 (1)  35 (27-43) |
| Employed as healthcare or laboratory worker, n (%) | 10/260 (4) |
| Travel within 14 days of admission, n (%) | 161/270 (60) |
| Co-existing conditions, n (%)  Hypertension  Chronic cardiac disease  Diabetes  Chronic lung disease  Asthma  Chronic liver disease  Chronic kidney disease  Prior TB  Active TB at time of admission | 25/270 (9)  0/270 (0)  2/270 (1)  3/270 (1)  5/270 (2)  0/270 (0)  0/270 (0)  2/270 (1)  0/270 (0) |
| Symptoms reported, n (%)  Cough  Fever  Headache  Rhinorrhea  Shortness of breath  Sore throat  Diarrhea  Night sweats Mildly symptomatic or asymptomatic, n (%)^a^ | 110/270 (41)  78/270 (29)  62/270 (23)  60/270 (22)  36/270 (13)  29/270 (11)  11/270 (4)  3/270 (1)  89/270 (33) |
| Duration of illness prior to hospitalization, days, median (IQR) ^b^ | 4 (2-7) |
| Received antibacterial or antimalarial agent prior to  admission, n (%) | 29/259 (11) |
| Vital signs  Temperature ≥38⁰C, n (%)  Heart rate, beats/min, median (IQR)  Respiratory rate, breaths/min, median (IQR)^c^  Systolic blood pressure, mmHg, median (IQR)  Oxygen saturation, %, median (IQR)  Oxygen saturation ≤ 94%, n (%)  Glasgow Coma Score, median (IQR)  Capillary refill time ≥3 seconds, n (%)  Unable to ambulate without assistance, n (%)  Mid-upper arm circumference, mm, median (IQR)^d^ | 15/270 (6)  85 (77-94)  18 (18-20)  120 (114-130)  97 (96-98)  41/270 (15)  15 (14-15)  0/268 (0)  11/270 (4)  21 (19-23) |
| Co-infections, n (%)  Malaria  HIV  New diagnosis of HIV  Taking ART prior to admission^e^  Microbiological TB^f^ | 4/270 (1)  26/270 (10)  11/26 (42)  10/15 (67)  0/21 (0) |
| Clinical Management, n (%)  Received oxygen therapy  Received antibacterial agent  Received HCQ or chloroquine  Received corticosteroids | 26/270 (10)  171/270 (63)  28/270 (10)  38/270 (14) |
| Patient outcomes, n (%)  Severe COVID-19  Died in-hospital  Transferred to national referral hospital  Karnofsky or Lansky score ≥ 80 at discharge | 48/270 (18)  0/270 (0)  1/270 (0.4)  266/267 (99) |

Abbrevations: IQR: interquartile range, HIV: human immunodeficiency virus, ART: anti-retroviral therapy, TB: tuberculosis, HCQ: hydroxychloroquine.

Legend: ^a^Defined as absence of reported cough, fever, headache, rhinorrhea, shortness of breath, sore throat, diarrhea, or night sweats; ^b^Known for 135 patients, ^c^Known for 255 patients, ^d^Known for 214 patients, ^e^Denominator of patients with known HIV prior to admission; ^f^Denominator of patients who underwent TB testing (urine TB-LAM or sputum Xpert MTB/RIF Ultra or smear).

**Table S2: Characteristics of patients with and without severe COVID-19 in phase 1**

| **Patient characteristic** | **Severe**  **illness**  **N=7**  **N (%)** | **Mild-moderate**  **illness**  **N=139**  **N (%)** |
| --- | --- | --- |
| Male sex, n (%) | 6/7 (86) | 126/139 (91) |
| Age, years, median (IQR) | 42 (28-60) | 34 (27-42) |
| Co-existing hypertension, n (%) | 0/7 (0) | 3/139 (2) |
| Symptoms reported, n (%)  Cough  Fever  Headache  Rhinorrhea  Shortness of breath  Sore throat  Diarrhea | 3/7 (43)  4/7 (57)  0/7 (0)  1/7 (14)  0/7 (0)  0/7 (0)  0/7 (0) | 33/139 (24)  30/139 (22)  23/139 (17)  27/139 (19)  2/139 (1)  12/139 (9)  4/139 (3) |
| Duration of illness prior to hospitalization, days, median (IQR)^a^ | 4 (3-7) | 3 (2-5) |
| Vital signs  Temperature ≥38⁰C, n (%)  Heart rate, beats/min, median (IQR)  Respiratory rate, breaths/min, median (IQR)^b^  Systolic blood pressure, mmHg, median (IQR)  Oxygen saturation, %, median (IQR)  Glasgow Coma Score, median (IQR)  Unable to ambulate without assistance, n (%)  Mid-upper arm circumference, mm, median (IQR)^c^ | 3/7 (43)  76 (70-89)  18 (18-22)  100 (95-110)  97 (89-98)  14 (14-15)  0/7 (0)  13 (13-21) | 5/139 (4)  81 (73-90)  18 (18-20)  120 (112-129)  97 (96-98)  15 (14-15)  0/139 (0)  20 (18-23) |
| Co-infections, n (%)  Malaria  HIV  Microbiological TB^d^ | 0/7 (0)  1/7 (14)  0/1 (0) | 3/139 (2)  17/139 (12)  0/15 (0) |
| Clinical Management, n (%)  Received oxygen therapy  Received antibacterial agent  Received HCQ or chloroquine  Received corticosteroids | 0/7 (0)  4/7 (57)  2/7 (29)  0/7 (0) | 0/139 (0)  71/139 (51)  26/139 (19)  0/139 (0) |

Abbrevations: IQR: interquartile range, HIV: human immunodeficiency virus, TB: tuberculosis, HCQ: hydroxychloroquine.

Legend: ^a^Known for 33 patients; ^b^Known for 144 patients; ^c^Known for 132 patients; ^d^Denominator of patients who underwent TB testing (urine TB-LAM or sputum Xpert MTB/RIF Ultra or smear).

**Table S3: Characteristics of patients with and without severe COVID-19 in phase 2**

| **Patient characteristic** | **Severe**  **illness**  **N=41**  **N (%)** | **Mild-moderate**  **illness**  **N=83**  **N (%)** |
| --- | --- | --- |
| Male sex, n (%) | 39/41 (95) | 54/83 (65) |
| Age, years, median (IQR) | 40 (35-50) | 33 (26-40) |
| Co-existing hypertension, n (%) | 5/41 (12) | 17/83 (20) |
| Symptoms reported, n (%)  Cough  Fever  Headache  Rhinorrhea  Shortness of breath  Sore throat  Diarrhea | 30/41 (73)  4/41 (10)  9/41 (22)  3/41 (7)  22/41 (54)  6/41 (15)  3/41 (7) | 44/83 (53)  21/83 (25)  30/83 (36)  29/83 (35)  12/83 (14)  11/83 (13)  4/83 (5) |
| Duration of illness prior to hospitalization, days, median (IQR)^a^ | 6 (3-7) | 4 (2-7) |
| Vital signs  Temperature ≥38⁰C, n (%)  Heart rate, beats/min, median (IQR)  Respiratory rate, breaths/min, median (IQR)^b^  Systolic blood pressure, mmHg, median (IQR)  Oxygen saturation, %, median (IQR)  Glasgow Coma Score, median (IQR)  Unable to ambulate without assistance, n (%)  Mid-upper arm circumference, mm, median (IQR)^c^ | 4/41 (10)  93 (83-105)  24 (19-24)  128 (117-133)  94 (92-97)  15 (15-15)  10/41 (24)  23 (20-24) | 3/83 (4)  89 (80-101)  18 (17-20)  122 (115-133)  97 (97-98)  15 (14-15)  1/83 (1)  22 (20-24) |
| Co-infections, n (%)  Malaria  HIV  Microbiological TB^d^ | 0/41 (0)  4/41 (10)  0/1 (0) | 1/83 (1)  4/83 (5)  0/4 (0) |
| Clinical Management, n (%)  Received oxygen therapy  Received antibacterial agent  Received HCQ or chloroquine  Received corticosteroids | 26/41 (63)  36/41 (88)  0/41 (0)  26/41 (63) | 0/83 (0)  60/83 (72)  0/83 (0)  12/83 (14) |

Abbrevations: IQR: interquartile range, HIV: human immunodeficiency virus, TB: tuberculosis, HCQ: hydroxychloroquine.

Legend: ^a^Known for 102 patients; ^b^Known for 111 patients; ^c^Known for 82 patients; ^d^Denominator of patients who underwent TB testing (urine TB-LAM or sputum Xpert MTB/RIF Ultra or smear).

**Table S4: Characteristics of patients with severe COVID-19 in phase-2 stratified by corticosteroid administration**

| **Patient characteristic** | **Administered**  **corticosteroids**  **N=26**  **N (%)** | **Not administered corticosteroids**  **N=15**  **N (%)** |
| --- | --- | --- |
| Male sex, n (%) | 24/26 (92) | 15/15 (100) |
| Age, years, median (IQR) | 43 (37-52) | 35 (28-42) |
| Symptoms reported, n (%)  Cough  Fever  Headache  Rhinorrhea  Shortness of breath  Sore throat  Diarrhea | 23/26 (88)  19/26 (73)  5/26 (19)  2/26 (8)  21/26 (81)  3/26 (12)  2/26 (8) | 7/15 (46)  4/15 (27)  4/15 (27)  1/15 (7)  1/15 (7)  3/15 (20)  1/5 (7) |
| Vital signs  Temperature ≥38⁰C, n (%)  Heart rate, beats/min, median (IQR)  Respiratory rate, breaths/min, median (IQR)^a^  Systolic blood pressure, mmHg, median (IQR)  Oxygen saturation, %, median (IQR)  Glasgow Coma Score, median (IQR)  Unable to ambulate without assistance, n (%)  Mid-upper arm circumference, mm, median (IQR)^b^ | 3/26 (12)  98 (90-114)  24 (20-24)  130 (120-137)  93 (92-94)  15 (14-15)  10/26 (38)  23 (20-24) | 1/15 (7)  80 (79-96)  17 (16-18)  126 (114-130)  98 (96-98)  15 (15-15)  0/15 (0)  22 (21-23) |
| Co-infections, n (%)  Malaria  HIV  Microbiological TB^c^ | 0/26 (26)  2/26 (8)  0/0 (0.0) | 0/15 (0)  2/15 (13)  0/1 (0.0) |
| Concomitant treatments, n (%)  Received oxygen therapy  Received antibacterial agent  Received HCQ or chloroquine | 25/26 (96)  26/26 (100)  0/26 (0) | 1/15 (7)  10/15 (67)  0/15 (0) |

Abbrevations: IQR: interquartile range, HIV: human immunodeficiency virus, TB: tuberculosis, HCQ: hydroxychloroquine.

Legend: ^a^Known for 28 patients; ^b^Known for 25 patients; ^c^Denominator of patients who underwent TB testing (urine TB-LAM or sputum Xpert MTB/RIF Ultra or smear).

**Supplemental Figures**

**Figure S1: Study Flow Diagram**


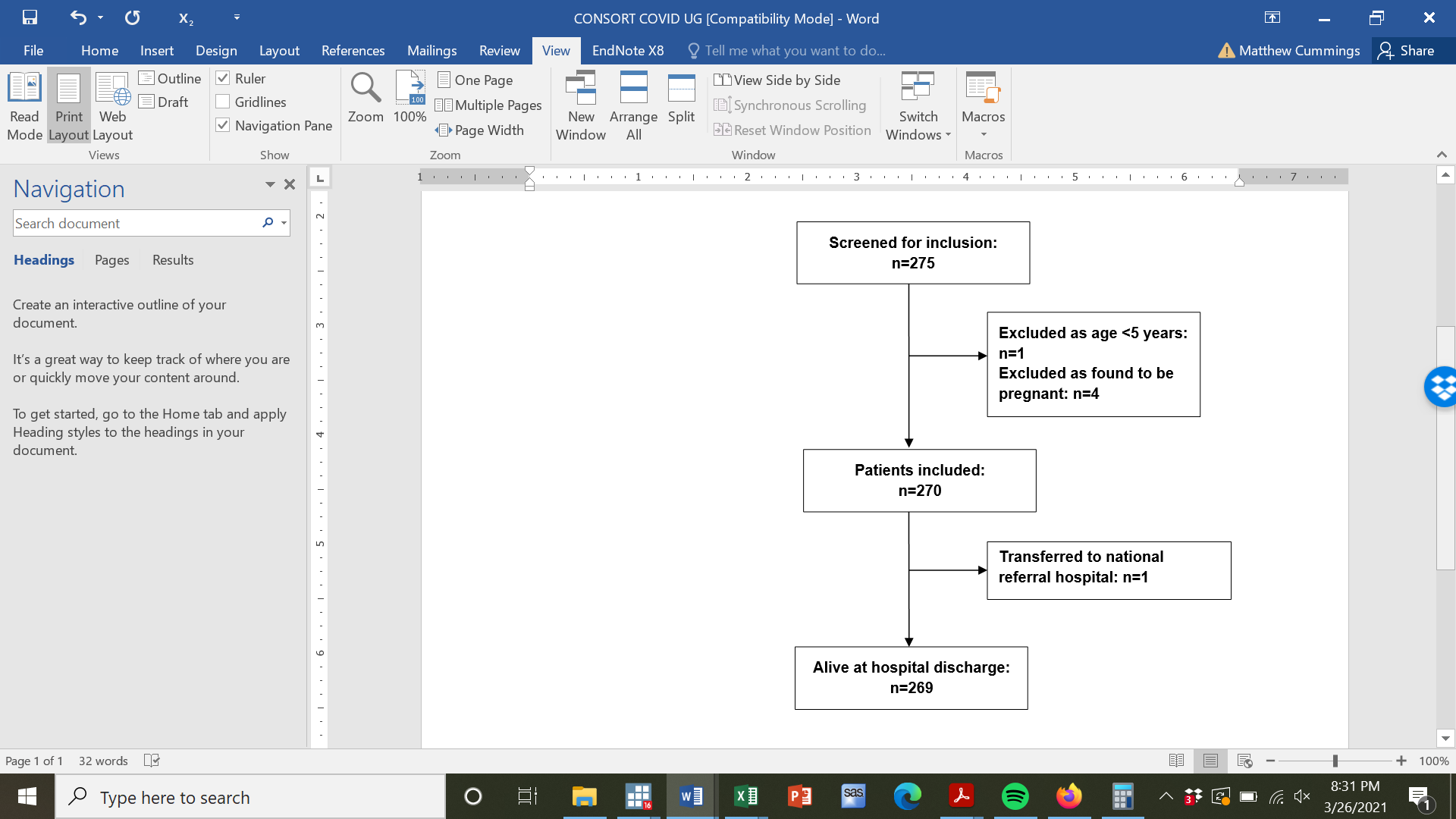

Supplement: Supplementary file 1 [file tpmd210551.SD1.docx]
